# Effects of Mitochondrial Uncoupling Protein 2 Inhibition by Genipin in Human Cumulus Cells

**DOI:** 10.1155/2015/323246

**Published:** 2015-08-19

**Authors:** Hongshan Ge, Fan Zhang, Dan Shan, Hua Chen, Xiaona Wang, Chao Ling, HaiTao Xi, Jianying Huang, ChunFang Zhu, Jeiqiang Lv

**Affiliations:** Reproductive Health Center, Second Affiliated Hospital of Wenzhou Medical University, 109 Xueyuan Road, Wenzhou, Zhejiang 325000, China

## Abstract

UCP2 plays a physiological role by regulating mitochondrial biogenesis, maintaining energy balance, ROS elimination, and regulating cellular autophagy in numerous tissues. But the exact roles of UCP2 in cumulus cells are still not clear. Genipin, a special UCP2 inhibitor, was added into the cultural medium to explore the roles of UCP2 in human cumulus cells. There were no significant differences in ATP and mitochondrial membrane potential levels in cumulus cells from UCP2 inhibiting groups as compared with the control. The levels of ROS and Mn-SOD were markedly elevated after UCP2 inhibited Genipin. However, the ratio of reduced GSH to GSSG significantly declined after treatment with Genipin. UCP2 inhibition by Genipin also resulted in obvious increase in the active caspase-3, which accompanied the decline of caspase-3 mRNA. The level of progesterone in culture medium declined obviously after Genipin treatment. But there was no significant difference in estradiol concentrations. This study indicated that UCP2 is expressed in human cumulus cells and plays important roles on mediate ROS production, apoptotic process, and steroidogenesis, suggesting UCP2 may be involved in regulation of follicle development and oocyte maturation and quality.

## 1. Introduction

Mammalian ovarian follicles are highly specialized structures that support the growth and development of oocytes. Bidirectional communication between the oocyte and its surround granulosa cells is essential for the growth and development of both follicle and oocyte [1]. There are two types of granulosa cells: the cumulus cells (CCs) and the mural granulosa cells (MGCs). The mural granulosa cells, located in the basal membrane of the follicles, keep in lesser close contact with oocyte due to the distance. Yet, CCs are closer to the oocyte and maintain a proximity relationship via transzonal processes and gap junctions with the oocyte, providing nutrients, maturation-enabling factors, and an optimal microenvironment to ensure successful maturation and further developmental competence [2, 3]. This close relationship between the oocyte and CCs implies that the CCs may serve as a biomarker for oocyte maturation and quality [4].

Providing a steady source of ATP plays a vital role in most cellular functions. Likewise, both in CCs and oocytes, energy in the form of ATP is thought to be critical for the processes of follicle growth, oocyte maturation, and fertilization and ensuing embryo development [5]. Mitochondria are the primary energy-generating system in most eukaryotic cells, including CCs and oocyte. Yet, unlike in most somatic cells where energy is produced via glucose, the oocyte is specialized with pyruvate as the main energy substrate [6, 7]. CCs have a special role to metabolize glucose into pyruvate, which then is transferred into oocyte [6]. Besides, the reactive oxygen species [8] is the inevitable byproduct of mitochondrial oxidative metabolism. But excessive amount of ROS, caused by mitochondria dysfunction or depletion of enzymatic antioxidant system, induces cellular oxidative stress (OS), promote apoptosis, and damage the quality of CCs and oocyte [9]. Together, these previous studies suggested that keeping a mitochondrial homeostasis in CCs is critical for oocyte metabolism and quality.

The uncoupling protein 2 (UCP2) belongs to the mitochondrial anion transporter superfamily that uncouple oxidative phosphorylation and regulate ATP synthesis [10]. The precise biochemical function of mitochondrial UCP2 is still a matter of debate. Accumulating literatures have showed that UCP2 plays a positive physiological role by regulating mitochondrial biogenesis, maintaining energy balance, keeping calcium homeostasis [11], ROS elimination [12], and regulating cellular autophagy [13] and, thereby, provides cellular protection and possibly anti-aging [14]. But some other studies that used inhibitors, knockdown, or mutagenesis methods indicated UCP2 might have many deleterious effects and were involved in pathogenesis of numerous diseases, such as cardiovascular diseases [15], type 2 diabetes mellitus [8], obesity [16], polycystic ovary syndrome (PCOS) [17], and various cancers [18].

Rousset et al. first reported that UCP2 is expressed in the female mouse reproductive tract, which was detected in ovary, oviduct, and uterus [19]. Roles of UCP2 in female reproductive tract were concerned by a few studies. The correlation of ovarian UCP2 with PCOS has been found by Liu et al. [17], and they observed that UCP2 in MGCs was strongly associated with the expression of P450scc protein, a key steroidogenic enzyme, suggesting that UCP2 may be involved in the pathogenesis of PCOS by altering androgen synthesis [17]. A previous study suggested that UCP2 was also closely related with the ROS generation and oocyte developmental potential [20]. The patients whose UCP2 was under the mean-level have higher ROS level in granulosa cells and impaired oocyte quality. In addition, authors also demonstrated that the expression of UCP2 in ovaries is correlated with female age. Levels of UCP2 from younger women were higher than that of advanced-age women [20]. The association between UCP2 expressing with fetal development in uterus and endometrium has also been confirmed [21, 22]. In the late stage of pregnancy, the expression of UCP2 in uterine would upregulate by several folds, suggesting UCP2 also plays some roles on fetal growth [22].

Yet, so far, the exact roles of UCP2 in CCs are still not clear. In this present study, to analyze the roles of UCP2 played in CCs, we introduced Genipin, a special UCP2 inhibitor to inhibit UCP2 functions. Genipin is extracted from the fruit of Gardenia and has been used to relieve symptoms of type II diabetes in traditional Chinese medicine [16]. Previous studies have shown that Genipin can decrease mRNA and protein level of UCP2, inhibit mitochondrial proton leak, promote mitochondrial membrane potential, and increase ATP synthesis [16]. The contents of ATP and mitochondrial membrane potential, levels of ROS and antioxidants, and apoptosis related protein caspase-3 are quantified in cultured CCs* in vitro*. In addition, the concentrations of estradiol and progesterone in culture medium are measured.

## 2. Materials and Methods

### 2.1. Patient Recruitment and Informed Consent

CCs were isolated from the infertile women who were treated with ICSI-ET at the Reproductive Medicine Center of the Second Affiliated Hospital of Wenzhou Medical University, Wenzhou, China. This study was approved by the Medical Ethical Committee of the hospital. Written informed consent was obtained from all couples participating in this study.

The long downregulation protocol was adopted for all the patients. All women were downregulated with a GnRH agonist on mid-luteal phase. When optimally downregulated, recombinant FSH (Merck Serono, Switzerland) was started on day 3 of the menstrual cycle. When one follicle size reached 18 mm or two follicles size reached 17 mm or three follicles size reached 16 mm, human chorionic gonadotropin (hCG, Merck Serono, Switzerland) was administered. At 36 hrs after administration of hCG, the follicles were aspirated through transvaginal ultrasound retrieval.

### 2.2. Isolation of Cumulus Cells and Cells Culture

CCs were separated mechanically from oocytes by mechanically pipetting for less than 1 min in 0.1% hyaluronidase solution and then transferred into 15 mL centrifuge tubes. The tubes were centrifuged at 1500 rpm for 5 min and the supernatant was decanted. The cells were washed twice with culture medium (DMEM/F12, Sigma-Aldrich, USA) containing 10% of fetal bovine serum (Sijiqing Bioen-gineering Material Co., Ltd., China) then cultured into 24-well culture plates. Cells were incubated at 37°C with 5.0% CO_2_ for 48 h with Genipin.

### 2.3. Cell Viability Measure

The cell count kit-8 (CCK, Beyotime, China) was used to quantitatively identify the cellular viability. The cultured CCs were rinsed twice with PBS, and then DMEM/F12 with 10% CCK-8 was added to these samples in a separate volume of 0.2 mL. After incubation for one hour, the absorbance was measured at 450 nm under a microplate reader (Bio-TEK, USA). Four parallel replicates of each sample at each time point were prepared during this cell viability assay.

### 2.4. Measurement of Mitochondrial Membrane Potential

JC-1 (5,5′,6,6′-tetrachloro-1,1′,3,3′-tetraethylbenzimidazoyl carbocyanine iodide, Anaspec Inc., USA) was a lipophilic cation and was used to assess the mitochondrial status in cells. CCs were incubated in culture medium with JC-1 at a concentration of 2.5 *μ*g/mL in a humidified incubator at 37°C, 5% CO_2_ for 20 min. CCs were washed twice at room temperature and centrifuged at 2000 rpm for 5 min. Then the cells were resuspended in 0.5 mL of PBS and analyzed them by BD FACSCalibur flow cytometry (BD, USA).

### 2.5. Measurement of ATP

The amount of ATP was measured by the ATP detection kit (Beyotime, China). CCs were lysed on ice with 100 *μ*L lysis buffer from ATP detection kit. After being centrifuged at 12,000 g for 5 min at 4°C, the supernatant was transferred to a new 1.5 mL tube for ATP test. Protein concentrations were determined by using a BCA Protein Assay Reagent Kit (Beyotime, China). The luminescence from a 100 *μ*L sample was assayed in a luminometer together with 100 *μ*L of ATP detection buffer from the ATP detection kit. The standard curve of ATP concentration was prepared from a known amount (1 nM–10 *μ*M).

### 2.6. Measurement of Intracellular ROS

The intracellular ROS of CCs was detected using DCHFDA. The CCs were incubated with 10 mM DCHF-DA in DMEM medium for 30 min at 37°C to allow cellular incorporation and then washed and resuspended in PBS. The ROS levels were detected by a microplate reader at 488 nm (Bio-TEK, USA).

### 2.7. Measurement of Lipid Peroxidation (MDA)

The lipid peroxidation in CCs was measured by Lipid Peroxidation MDA Assay Kit (Beyotime, China). CCs were lysed and homogenized and then centrifuged at 20000 ×g at 4°C for 10 min, and then the supernatants were collected. Protein concentrations were determined by using a BCA Protein Assay Reagent Kit (Beyotime, China). A 200 *μ*L of thiobarbituric acid (TBA) reagent was added into 100 *μ*L of the suspension in 96-well plates. The mixture was treated in a boiling water bath for 15 min. After cooling, the suspension was centrifuged at 1,000 ×g at room temperature for 10 min and the supernatant was separated, then the absorbance was measured at 530 nm under a microplate reader (Bio-TEK, USA).

### 2.8. Measurement of Mn-SOD Activity

Superoxide dismutase (SOD) activity was measured using a Cu-Zn/Mn-SOD assay kit (WST) (Beyotime, China). Briefly, the Mn-SOD activity was measured by reduction rate inhibitions of 2-(4-iodophenyl)-3-(4-nitrophenyl)-5-(2,4-disulfophenyl)-2H-tetrazolium and monosodium salt (WST-8) adding Cu-Zn-SOD inhibitor A and inhibitor B to inactivate Cu-Zn-SOD activity. Mn-SOD activity was expressed as units per mg of protein (one unit was defined as the amount of enzyme hat inhibited WST-1 reduction by 50%). Protein concentrations were determined by using a BCA Protein Assay Reagent Kit (Beyotime, China). The sample was diluted with PBS by 1 : 1, and then mix with the working solution in a 96-well plate. After incubated at 37°C for 20 mins, the absorbance was measured at 450 nm under a microplate Reader (Bio-TEK, USA).

### 2.9. Colorimetric Determination of Reduced and Oxidized Glutathione

The reduced GSH and oxidative GSH (GSSG) concentrations in CCs were measured by using a GSH/GSSG kit (Beyotime, China). The GSSG and GSH standards were prepared in 5% metaphosphoric acid. Cell samples were prepared in 5% metaphosphoric acid with or without 1-methyl-2-vinyl-pyridium trifluoromethane sulfonate, a GSH-specific scavenger. Ellman's reagent (5,5-dithiobis-2-nitrobenzoic acid) reacts with GSH to form a product with an absorption maximum at 412 nm. GSSG was determined using glutathione reductase to reduce GSSG to GSH followed by reaction with Ellman's reagent.

### 2.10. Measurement of Estradiol and Progesterone Concentrations

The CCs culture medium was collected and stored at −20°C until the progesterone or estradiol was assay. Progesterone concentrations in the medium were measured by a specific radioimmunoassay (RIA). The sensitivity of the assay was 3.5 pg/tube, and the intra-assay and interassay coefficients of variation were 8.7 and 6.3%, respectively. Estradiol concentrations in the medium were measured by enzyme-linked immunosorbent assay (ELISA) kit (Neogen, Lexington, KY), and the intra-assay and interassay coefficients of variation were less than 4%.

### 2.11. Real-Time PCR

At the beginning of the experiment, cells were harvested at the indicated time points for the extraction of RNA, and 800 *μ*g of total RNA was reverse-transcribed into cDNA (TRizol, Invitrogen). To measure mRNA expression of CX 43 and caspase-3, RT-PCR using the SYBR green (Takara) was used according to the manufacturer's instructions. The following primers were used: caspase-3: GCCGTGGTACAGAACTGGACT, GCACAAAGCGACTGGATGAA. *β*-actin was used as an internal reference and its primers: TGACGTGGACATCCGCAAAG and CTGGAAGGTGGACAGCGAGG.

### 2.12. Western Blotting

Proteins from CCs were isolated with RIPA lysis buffer (20 *μ*L) (Beyotime, China). The mixture was centrifuged at 12000 g for 20 min at 4°C and the supernatant transferred into 1.5 mL Eppendorf tube. Protein concentrations were determined using a BCA Protein Assay Reagent Kit (Beyotime, China). Caspase-3 protein was quantified by western blotting. The primary antibody to caspase-3 was used at a concentration of 1 : 1000 (Cell Signaling Technology, USA). The mouse monoclonal antitubulin antibody (Beyotime, China) was used for normalization. Secondary antibodies were conjugated with horseradish peroxidase, and the signals were detected using SuperSignal West Pico (Pierce, USA).

### 2.13. Statistical Analysis

All experiments were performed in triplicate. Data are presented as the mean ± SE. The results were analyzed with Student's *t*-test or by one-way* ANOVA*. *P* values < 0.05 were considered as significant using SPSS 13.0 software (Chicago, IL, USA).

## 3. Results

### 3.1. Effects of UCP2 Inhibition by Genipin on Cellular Viability

**Figure 1:**
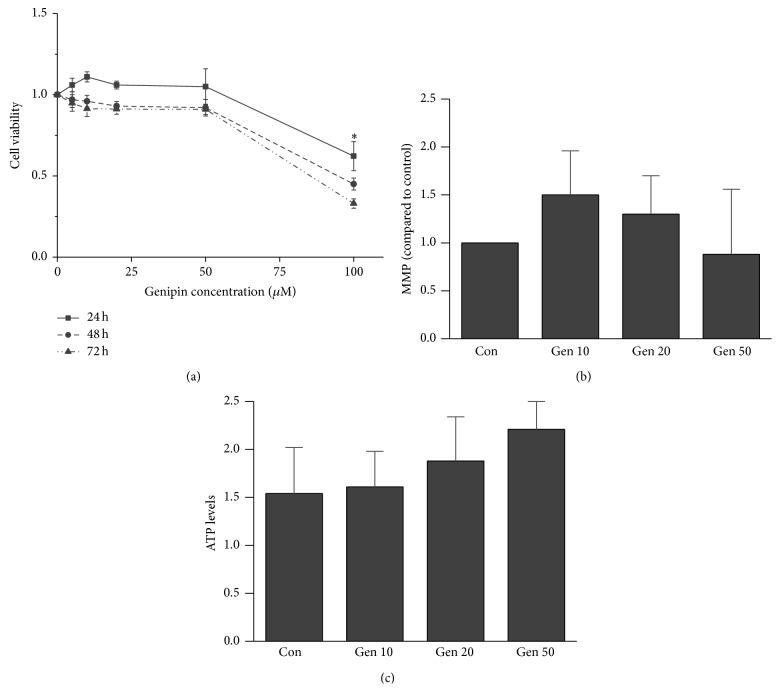
Effects of UCP2 inhibited by Genipin on cell viability, contents of ATP, and mitochondrial membrane potential (MMP). No significant differences were found in cell viability, contents of ATP, and mitochondrial membrane potential of CCs from UCP2 inhibited by Genipin as compared with their controls. Con means control and MMP means mitochondrial membrane potential.

A CCK8 assay was used to determine the appropriate concentration of Genipin choice in the study, which would not result in significant cytotoxicity on CCs. As shown in Figure 1(a), cultivating CCs in Genipin concentrations 0, 10, 20, and 50 *μ*M, there were not significantly impacts on cellular morphology, size, and number. But the cell viability and cellular morphology were changed significantly when the Genipin concentration reached 100 *μ*M (*P* < 0.05, Figure 1(a)). So, the 50 *μ*M Genipin was choice in this study.

### 3.2. Effects of UCP2 Inhibition by Genipin on Activity of OXPHOS

To determine the effects of UCP2 on the activity of OXPHOS, the ATP content and levels of mitochondrial membrane potential in CCs were measured. In Figures 1(b) and 1(c), there were no significant differences in both ATP contents and mitochondrial membrane potential levels were found in all Genipin treated groups as compared with the control (*P* > 0.05).

### 3.3. Effects of UCP2 Inhibition by Genipin on Oxidative Stress

**Figure 2:**
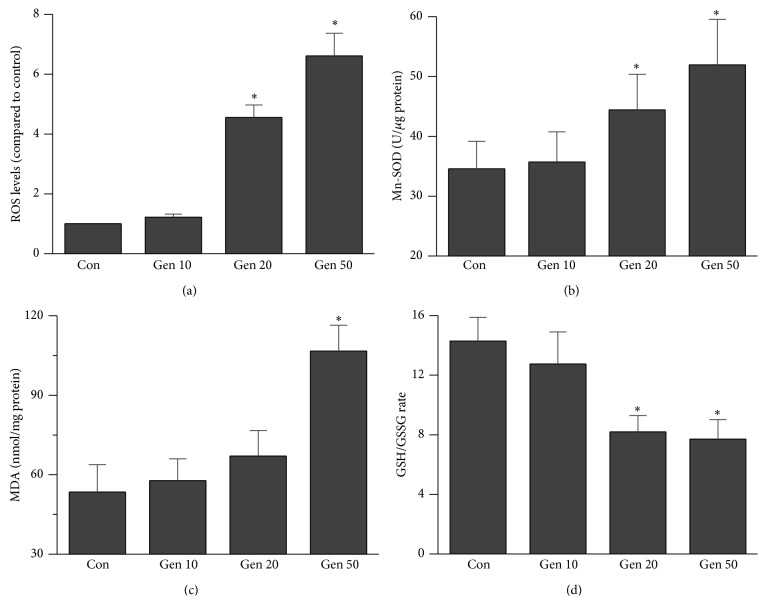
Effects of UCP2 inhibited by Genipin on oxidative stress. (a) The level of ROS in CCs has an increasing tendency after UCP2 inhibiting. (b) The Mn-SOD activity has an increasing tendency after UCP2 inhibiting. (c) The MDA level has an increasing tendency after UCP2 inhibiting. (d) The ratio of reduced GSH to GSSG has a decline tendency after UCP2 inhibiting. Con means control, asterisks indicate significant difference between control and treatment group at *P* < 0.05.

**Figure 3:**
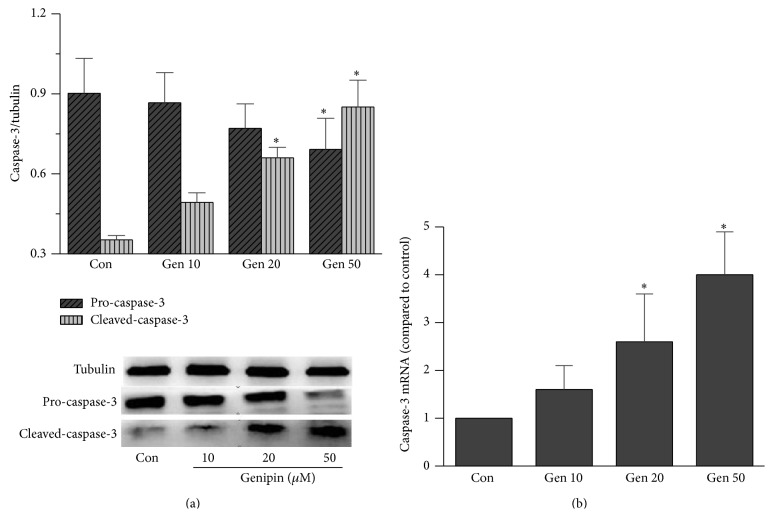
Effects of UCP2 inhibited by Genipin on CCs apoptosis. (a) The caspase-3 protein was quantified by Western blotting. The proactive caspase-3 declined obviously, and the active caspase-3 markedly increased after UCP2 inhibition. (b) Caspase-3 gene was quantified by RT-PCR, which markedly increase after UCP2 inhibition. Con means control, asterisks indicate significant difference between control and treatment group at *P* < 0.05.

To determine effects of UCP2 on cellular redox status in CCs, Mn-SOD activity and the levels of the ROS, ratio of GSH/GSSG, and MDA concentration were evaluated, respectively. The intracellular ROS generation in CCs was markedly elevated after CCs were treated with 20 and 50 *μ*M Genipin (*P* < 0.05, Figure 2(a)), which might feedback induce an increase of Mn-SOD in 20 and 50 *μ*M Genipin treated groups (*P* < 0.05, Figure 3(b)). And obviously decreases were detected in the ratio of reduced GSH to GSSG in 20 and 50 *μ*M Genipin treated groups too (*P* < 0.05, Figure 2(d)). In contrast, the MDA concentration increased significantly after being treated with 50 *μ*M Genipin as compared with control (*P* < 0.05, Figure 2(c)).

### 3.4. Effects of UCP2 Inhibition by Genipin on CCs Apoptosis

To evaluate the effects of UCP2 on CCs apoptosis, the protein and gene of caspase-3, an executioner caspase, were, respectively, assessed. Two types caspase-3 protein were observed by WB, a proactive caspase-3 (32 kD) and an active caspase-3 (17 kD). UCP2 inhibition resulted obviously in an increase in expression of the active caspase-3, which was associated with the decline of proactive caspase-3 in 20 and 50 *μ*M Genipin treated groups as compared with control (*P* < 0.05, Figure 3(a)). The expression of mRNA of caspase-3 has the same tendency as showed in caspase-3 protein (*P* < 0.05, Figure 3(b)).

### 3.5. Effects of UCP2 Inhibition by Genipin on Steroidogenesis

**Figure 4:**
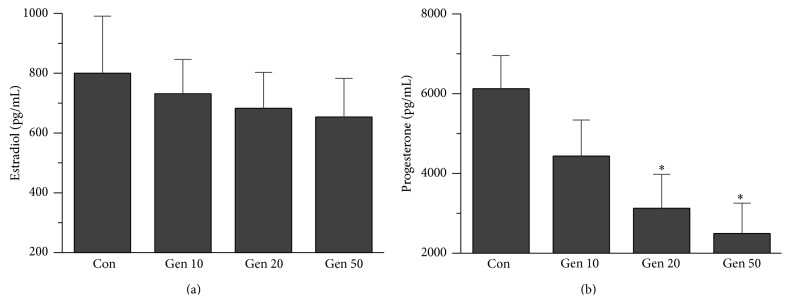
Effects of UCP2 inhibited by Genipin on steroidogenesis. (a) A slight decline of concentrations of estradiol in culture medium was found, but no significant difference was detected between UCP2 inhibited by Genipin and the control. (b) The level of progesterone in culture medium decline obviously after Genipin treated. Con means control, asterisks indicate significant difference between control and treatment group at *P* < 0.05.

To evaluate the effects of UCP2 on steroid hormone secretion of CCs* in vitro*, the concentrations of estradiol and progesterone in culture medium were measured. A decline of concentrations of estradiol was found, but no significant difference was detected in all Genipin treated groups as compared with control (*P* > 0.05, Figure 4(a)). On the contrary, the level of progesterone in culture medium has a decline tendency after Genipin treatment, and significant differences were detected in 20 and 50 *μ*M Genipin treated groups (*P* < 0.05, Figure 4(b)).

## 4. Discussion

Numerous researches observed that UCP2 plays key roles in regulation of ATP production and maintaining cellular energy balance. Regulating UCP2 expression by UCP2 inhibitors or analogues, such as FCCP or DNP, can alter cellular ATP contents in many cell types [16, 23]. However, in this study, UCP2 inhibition by Genipin does not lead to a visible change in ATP levels in CCs. This is consistent with several previous studies, in which overexpression of UCP2 did not alter the ATP contents of mouse brain cell [24]. No significant alteration in activity of OXPHOS was detected after UCP2 suppression, implying that regulatory functions of ATP generation or maintaining the energy balance might not be the primary role of UCP2 in human CCs, where it may play roles priority in reducing ROS generation, promoting apoptosis, or some others. Besides, some previous studies demonstrated that, apart from OXPHOS, other pathways, including the glycolysis and the pentose phosphate pathway (PPP), are confirmed to be involved in ATP production in CCs [25], which may help explain our results.

The oxidative damage of CCs or in follicular fluid is observed with a negative association with oocyte maturation, fertilization, and ensuing embryo developmental potential, as well as IVF/ICSI results [9, 26, 27]. A previous study suggested that overgeneration of ROS, caused by mitochondria dysfunction or depletion of enzymatic antioxidant system, could inhibit steroidogenic enzymes activity in MGCs and CCs [28]. Mild mitochondrial uncoupling has been proposed as a mechanism to decrease ROS generation, in which UCP2 inhibiting or overexpression can markedly alter ROS production in numerous tissues [24, 29]. In CCs, we found the same effects. UCP2 inhibited by 20 and 50 *μ*M Genipin markedly increase mitochondrial ROS production. High ROS production often leads to compensatory upregulation of antioxidant responses, which can also be observed in this study. UCP2 suppression increases ROS generation leading to increasing Mn-SOD activity feedback. However, the ratio of GSH/GSSG reduces obviously and is concomitant with an increase of the MDA level, suggesting that UCP2 suppression can aggravate the oxidative injury in CCs. Our result is consistent with a former research in MGCs [20], which suggested there is a UCP2-ROS mutual-regulation system in granulosa cells.

Correlation of UCP2 with apoptosis has also been observed in many type cells, where it endows an antiapoptotic property [30]. Inducing UCP2 overexpression can inhibit apoptosis process [31]. In contrast, inhibition or knockdown UCP2 can promote apoptosis [30]. In our studies, we observed that treatment with 20 and 50 *μ*M Genipin, the active form of caspase-3, increased significantly, suggesting UCP2 suppression by Genipin promotes apoptosis in CCs. Our results also support a previous observation in cultured MGCs, which UCP2 inhibited by exogenous ATP, resulting in an increase in the apoptotic rate and a decrease of the cellular antioxidant capability [32].

For steroidogenesis, the initial step occurs within the mitochondrion. The intact mitochondrial structure and functions are the requirement of steroidogenesis [33]. So, in this study, it is not unexpected that UCP2 inhibition by Genipin can affect steroid hormone levels in CCs culture medium, especially the progesterone level, a hormone essential for implantation and maintenance of pregnancy in mammals. Roles of UCP2 regulating steroidogenesis have been observed in a former study. UCP2 can inhibit androgen synthesis in MGCs of PCOS patience [17], and inducing UCP2 overexpression* in vitro* would increase testosterone synthesis in both normal and PCOS granulosa cells.

In conclusion, our results demonstrated that* in vitro* UCP2 plays important roles on mediate ROS production, apoptotic process, and steroidogenesis. UCP2 inhibition results in increase of cellular ROS generation, disturbs cellular redox status, and induces apoptosis, as well as impairing progesterone production, but does not significantly affect the activity of OXHPOS. But we have to recognize that, apart from UCP2 inhibiting, Genipin might have some other nonspecific effects, which might affect our conclusions. So, further study is required to use other methods such as UCP2-siRNA to explore the impacts of UCP2 on CCs and its impact on oocyte maturation and fertilization and ensuing embryonic development potential* in vitro* and* in vivo*.
